# Physical Activity and Eating Habits Are Related to Chronic Disease in the Basic Livelihood Security Program

**DOI:** 10.3390/nu17030462

**Published:** 2025-01-27

**Authors:** Seongryu Bae, Hyuntae Park

**Affiliations:** 1 Department of Health Sciences, The Graduate School of Dong-A University, Busan 49315, Republic of Korea; bae.seongryu@gmail.com; 2 Digital Healthcare Institute, Dong-A University, Busan 49315, Republic of Korea

**Keywords:** basic livelihood security, income, dietary habit, physical activity, older adults

## Abstract

Objectives: Chronic diseases are a significant public health issue, especially for socioeconomically vulnerable population groups. The purpose of this study is to compare the prevalence of chronic diseases in people receiving and not receiving BLS and to determine the prevalence of chronic diseases according to the physical activity and dietary habits of people receiving BLS. Methods: Data were derived from the sixth to ninth waves (2014–2022) of the Korea National Health and Nutrition Examination Survey (KNHANES), focusing on 15,041 participants aged 65 and older. Demographic characteristics, dietary intake, physical activity, and chronic disease status were assessed. Multivariate logistic regression analysis was used to calculate odds ratios for chronic diseases according to physical activity and dietary habits. Results: The BLS group exhibited higher prevalence rates of hypertension and diabetes, along with lower dietary intake of energy, protein, fat, carbohydrates, dietary fiber, and vitamin C, compared to the non-BLS group. A below-average intake of energy and carbohydrates was associated with increased odds of hypertension and diabetes, particularly in the BLS group. For dietary fiber, a significant association with diabetes was found only in the BLS group. Sedentary behavior exceeding 9 h per day was linked to higher odds of chronic diseases in both groups, with stronger associations in the BLS group. Limited walking frequency (less than 1 day per week) further exacerbated risks. Conclusions: BLS recipients demonstrated higher chronic disease prevalence, poorer dietary habits, and more sedentary behavior compared to non-recipients. The associations between lifestyle factors and chronic diseases were generally more substantial in the BLS group, suggesting the need for targeted interventions to improve dietary quality and physical activity patterns in this vulnerable population.

## 1. Introduction

Chronic diseases, including diabetes, cardiovascular diseases, hypertension, and obesity, are major contributors to the global public health burden. Collectively, they account for the majority of deaths and disability-adjusted life years (DALYs) lost worldwide. The World Health Organization (WHO) estimates that chronic diseases are responsible for approximately 71% of global deaths annually, with cardiovascular diseases alone contributing to over 17.9 million deaths [1]. The interaction between dietary habits and physical activity profoundly impacts the onset and progression of chronic diseases. Inadequate physical activity is strongly associated with obesity and metabolic syndrome, while unhealthy diets increase the risk of type 2 diabetes, hypertension, and cardiovascular diseases [2]. These risk factors often co-occur, creating a cycle of poor health outcomes that disproportionately affects low-income individuals. The clustering of these risk factors leads to multiple chronic conditions, exacerbating health inequities.

Low-income populations bear a disproportionate burden of chronic diseases due to the interplay of behavioral, environmental, and structural factors. Key drivers include limited access to healthcare, lower health literacy, inadequate nutrition, and higher levels of exposure to environmental risks such as pollution and urban crowding [3,4]. Structural inequalities, including socioeconomic disparities and systemic discrimination, further exacerbate these health inequities [5]. Dietary patterns and physical inactivity are significant behavioral risk factors contributing to the prevalence of chronic diseases in low-income populations. Ultra-processed and high-calorie foods are often more accessible and affordable in economically disadvantaged communities, promoting obesity and metabolic syndromes [6]. Moreover, food deserts are areas with limited access to affordable and nutritious food and contribute to poor dietary practices, compounding health risks [7]. Similarly, urban infrastructure in low-income areas often lacks spaces conducive to physical activity, such as parks or recreational facilities [8].

Environmental factors, including exposure to pollution and limited access to clean water and sanitation, also disproportionately affect low-income populations, increasing the risk of conditions such as cardiovascular diseases and respiratory illnesses [9]. Interventions targeting these disparities require a multifaceted approach, emphasizing health education, equitable healthcare access, and structural reforms to address social determinants of health. Policies promoting affordable healthy foods, community-based health programs, and investments in sustainable urban infrastructure are essential to mitigating the disproportionate burden of chronic diseases in low-income populations.

South Korea’s Basic Livelihood Security (BLS) system is a comprehensive public assistance program designed to support low-income individuals and families by ensuring a minimum standard of living. Established under the National Basic Livelihood Security Act in 2000, it replaced earlier welfare models to provide more systematic and equitable support. People receiving BLS are targeted at people with extremely low incomes. Thus, this group is particularly vulnerable to adverse health outcomes due to socioeconomic constraints, limited access to healthcare services, and high levels of exposure to health risks. According to a study by Chung et al. (2021), BLS recipients were more likely to have metabolic syndrome than non-recipients, highlighting the combined impact of poverty and health risk factors [10]. Research indicates that metabolic syndromes are more common in the BLS group than in the non-BLS group. In the non-BLS and BLS groups, an increased prevalence of MS was associated with high caloric intake, low breakfast frequency, low nutritional awareness, low walking frequency, and low levels of strength exercise [11]. Sedentary behavior is another critical issue among BLS recipients. Park et al. (2020) found that individuals receiving BLS assistance had higher sedentary time and lower physical activity levels, contributing to increased risks of obesity, diabetes, and cardiovascular diseases [12].

Although studies on the lifestyle patterns and characteristics of people receiving BLS and their health outcomes are being reported, it is still insufficient to study the roles of nutrition and physical activity in relation to receiving BLS and chronic diseases. The purpose of this study is to compare the prevalence of chronic diseases in people receiving and not receiving BLS and to determine the prevalence of chronic diseases according to the physical activity and dietary habits of people receiving BLS.

## 2. Materials and Methods

### 2.1. Data and Study Population

This study examined the health and nutritional status of the Korean population using data collected from the sixth through ninth waves (2014–2022) of the Korea National Health and Nutrition Examination Survey (KNHANES), conducted by the Korean Ministry of Health and Welfare [13]. The KNHANES is widely recognized for its role in shaping Korea’s public health strategies and improving the overall health of its citizens. It also serves as a valuable resource for researchers and policymakers globally. Researchers can access this dataset via the official website https://knhanes.kdca.go.kr/knhanes/main.do (accessed on 20 September 2024). We analyzed 15,041 of the 68,023 individuals aged 65 and older who participated in the KNHANES. Using a questionnaire, we included people who had previously received BLS or were currently receiving BLS in the BLS group. We included people who had not received BLS in the non-BLS group. The data used in this study were approved by the Institutional Review Board of the Korean Centers for Disease Control and Prevention (2013-07CON-03-4C, 2013-12EXP-03-5C, 2018-01-03-P-A, 2018-01-03-C-A, 2018-01-03-2C-A, 2018-01-03-5C-A, 2018-01-03-4C-A), and written informed consent was obtained from all participants.

### 2.2. Participants Characteristics

Education status (to elementary, middle, or high school, or above college), marital status (with spouse or not married), smoking history (never and past or current), hypertension (yes or no), and diabetes mellitus (yes or no) were assessed using self-reported questionnaires. Blood pressure was measured two times on the right arm and the average value was used. Body mass index (BMI) was calculated as body weight (kg) divided by the square of height (m).

### 2.3. Chronic Disease

The present study surveyed the diagnosis of hypertension and diabetes, common chronic diseases. Participants reported whether they had been diagnosed with hypertension and diabetes mellitus by a healthcare provider. Blood pressure was measured to determine hypertension and blood samples were collected and analyzed to determine fasting blood glucose and hemoglobin A1c. Blood samples were taken from the participant’s vein. The samples were immediately processed, refrigerated, and transported to the central laboratory of the Seegene Medical Foundation in Seoul. Total cholesterol, triglycerides, and fasting plasma glucose were measured using the enzymatic method, all on an automated analyzer (Hitachi Automatic Analyzer 7600, Tokyo, Japan).

### 2.4. Dietary Habits

Dietary intake data were collected through face-to-face interviews conducted by trained interviewers and dietitians. The 24 h recall method was employed to assess participants’ daily dietary intake. Participants were asked to recall all foods and beverages consumed the previous day, including portion sizes and cooking methods. Daily intakes of energy and nutrients, including total energy, protein, fat, carbohydrates, dietary fiber, and vitamin C, were calculated using Can-Pro 2.0 nutrient intake assessment software (Korean Dietetic Association, Seoul, Republic of Korea) [14]. Can-Pro 2.0 is widely used in Korea for nutritional research, clinical practice, and public health studies. The software incorporates a comprehensive database of Korean foods, including detailed nutrient profiles such as macronutrients (carbohydrates, proteins, and fats), vitamins, minerals, and dietary fiber. The data collected were used to analyze dietary patterns and nutrient consumption.

### 2.5. Physical Activity

To assess frequency of physical activity, participants were asked, “How much time per day do you spend sedentary?” Participants were categorized into groups based on their sedentary time: 5 h or less, 6–8 h, 9–11 h, and 12 h or more per day. In addition, participants were categorized into three groups: those who walked more than 5 days a week, 2–4 days a week, and less than 1 day a week.

### 2.6. Statistical Analysis

Two-sample *t*-tests and chi-square (χ^2^) tests were conducted to examine differences in continuous and categorical variables and to compare demographic and clinical characteristics between non-BLS and BLS groups. In the χ^2^ test, the statistical significance of the cells in the table was examined using residual analysis. If the adjusted standardized residual was greater than 1.96, the cell was considered to contain significantly more people than expected, and if it was lower than −1.96, the cell was considered to contain significantly fewer people than expected. Multivariate logistic regression analysis was used to calculate odds ratios (OR) and 95% confidence intervals (CI) for the prevalence of hypertension, dyslipidemia, and diabetes according to physical activity and dietary habits, respectively. In addition, we adjusted for sex, education status, and smoking history as confounding factors. The non-BLS group and the groups with above-average intake of each nutrient were used as references. The nutrient intake for each was divided based on the average for people aged 65 and older from the KNHANES. Analyses were conducted using SPSS version 28.0 (IBM Corp., Armonk, NY, USA). Statistical significance was set a priori at *p* < 0.05.

## 3. Results

### 3.1. Demographic Characteristics of Participants

Table 1 shows the demographic characteristics of the non-BLS and BLS groups. There was a higher proportion of females in the BLS compared to the non-BLS group. Systolic and diastolic blood pressure were slightly higher in the BLS group. In addition, there was a higher prevalence of hypertension and diabetes mellitus in the BLS group. The BLS group had a higher percentage of individuals with elementary or lower education. More individuals in the non-BLS group were married than those in the BLS group. The BLS group showed lower intake levels of energy, protein, fat, carbohydrates, dietary fiber, and vitamin C. In terms of the biochemical parameters, fasting blood glucose, hemoglobin A1c, and triglycerides were higher in the BLS groups. The BLS group was likely to spend more time being sedentary and walked less regularly.

### 3.2. Association of Chronic Disease and Dietary Habits

Table 2 shows the association between nutrient intake and prevalence of hypertension using multivariate logistic regression analysis. A below-average energy consumption and carbohydrate intake were associated with significantly higher odds of hypertension in the BLS group, but no significant association was observed in the non-BLS group. Protein, dietary fiber, and vitamin C were associated with a higher risk of hypertension in the BLS group, regardless of whether they consumed more or less than average. For people with below-average fat intake, both groups had significantly increased odds of hypertension, with the OR being higher in the BLS group than in the non-BLS group.

Results of the association analysis of nutrient intake and diabetes prevalence are shown in Table 3. In both groups, below-average energy and fat intake were associated with a higher prevalence of diabetes; however, the OR was higher in the BLS group than in the non-BLS group. For protein, carbohydrates, and vitamin C, a below-average intake was associated with higher odds of diabetes in the non-BLS and the BLS groups. In addition, it was found in the BLS group that people with above-average protein, carbohydrates, and vitamin C intake also had a higher prevalence of diabetes. Lastly, regarding dietary fiber, below-average intake was not associated with increased odds in the non-BLS group, whereas it was significantly associated with increased odds in the BLS group.

### 3.3. Association of Chronic Disease and Physical Activity

The multivariate logistic regression analysis demonstrated the associations between physical activity and the prevalence of hypertension (Table 4). A sedentary time of less than 5 h was not associated with a prevalence of hypertension in the BLS group. Individuals spending 6–8 h and 9–11 sedentary per day had significantly higher odds of hypertension in both the non-BLS and the BLS group. Similarly, those with 9–11 h of sedentary time showed increased odds of hypertension. Individuals spending 12 or more sedentary hours per day had the highest odds of hypertension, with ORs of 1.240 for non-BLS and 1.542. In addition, the odds of hypertension were higher for the BLS group than the non-BLS group. Regarding the number of walking days per week, individuals walking 2–4 days per week showed no significant association in the non-BLS group, while the BLS group showed significantly higher odds. For those walking one day or less per week, the odds of hypertension were significantly elevated in both groups.

The association between physical activity and diabetes prevalence was similar to that of hypertension prevalence (Table 5). In the non-BLS group, sedentary behavior lasting 9–11 and 12 or more hours per day was associated with an increased risk of diabetes mellitus, whereas sitting for 6–8, 9–11, and 12 or more hours per day was associated with an increased risk of diabetes in the BLS group. The number of days per week walking has also been shown to be associated with diabetes prevalence. In the non-BLS group, walking less than one day per week was associated with diabetes prevalence, whereas, in the BLS group, walking two to four days per week and less than one day per week was associated with diabetes prevalence.

## 4. Discussion

This study highlights significant differences in demographic, dietary, physical activity, and biochemical characteristics between the non-BLS and BLS groups, which may explain the high prevalence of hypertension and diabetes observed in the BLS group. These results are consistent with previous findings that emphasize the importance of socioeconomic status and lifestyle for health outcomes [4,15]. The higher proportion of people educated at the primary school level or below in the BLS group may reflect socioeconomic inequalities related to limited access to health information, low health literacy, and poor resources for disease prevention [16].

Nutritional imbalances were also found, with the BLS group consuming less energy, protein, fat, carbohydrates, dietary fiber, and vitamin C. Poor intake of dietary fiber and essential nutrients is associated with an increased risk of obesity and insulin resistance, and poor nutritional intake is well known as a risk factor for cardiovascular and metabolic diseases [17,18]. According to one study, low-income people eat a lot of high-fat potatoes and meat, while high-income people eat a lot of low-fat dietary fiber and plant-based protein [19]. Similar findings have been observed in other studies exploring different income groups’ dietary habits. For instance, research in the United States highlights that low-income individuals are more likely to consume energy-dense, nutrient-poor foods such as processed meats, fried foods, and sugary beverages due to their affordability and accessibility. In contrast, high-income groups tend to incorporate more fruits, vegetables, whole grains, and plant-based proteins into their diets, which are often costlier and perceived as healthier options [20]. Another study in the United Kingdom revealed that individuals with lower socioeconomic status usually face barriers to accessing diverse food options. Their diets were characterized by lower intakes of fiber, vitamins, and minerals and higher consumption of saturated fats and refined carbohydrates. This dietary pattern is associated with increased risks of obesity, diabetes, and cardiovascular diseases [21]. In addition, a study focusing on dietary patterns in Brazil found that households with lower incomes allocated a significant proportion of their budget to staple foods like rice, beans, and oils. In contrast, higher-income households spent more on fresh produce, lean meats, and dairy products. The disparity in food choices contributes to significant nutritional differences, and low-income households were more likely to experience deficiencies in essential nutrients [22]. Across a wide range of population groups, there is a consistent trend in the effect of income on food choice and nutrition. Low-income people have limited food choices, which inevitably leads to nutritional imbalances.

Biochemical parameters further underscored the metabolic burden in the BLS group, as evidenced by higher fasting blood glucose, hemoglobin A1c, and triglyceride levels. These markers of poor glycemic control and dyslipidemia are strongly associated with an elevated risk of diabetes mellitus and cardiovascular disease [23,24]. Lifestyle factors also played an important role, with the BLS group exhibiting more sedentary behavior and fewer regular walking days. Sedentary behavior has been identified as an independent risk factor for diabetes and cardiovascular disease, regardless of overall physical activity levels [25,26]. Furthermore, a recent study by Matthews et al. (2020) focused on sedentary behavior in older adults [27]. The study found that those with higher sedentary time had significantly worse cardiovascular health metrics compared to peers with less sedentary behavior. This relationship persisted even when controlling for other lifestyle factors, including regular exercise.

The results of the logistic analysis of hypertension and nutritional status showed that below-average energy and carbohydrate intake was significantly associated with an increased probability of hypertension in the BLS group but not in the non-BLS group. This suggests that energy intake and carbohydrate deficiencies disproportionately affect individuals with low socioeconomic status. Nutritional deficiencies in the elderly are often linked to a lack of essential micronutrients such as potassium, magnesium, and calcium, which play an important role in blood pressure regulation [28]. Potassium deficiency, for instance, has been shown to impair vascular function, leading to increased blood pressure. Research indicates that insufficient potassium intake can promote vascular calcification, further exacerbating cardiovascular diseases, including hypertension [29]. In addition, chronic carbohydrate deficiency can increase oxidative stress and inflammation, further elevating the risk of hypertension in older adults [30]. A review of the relationship between dietary nutrients and oxidative stress in chronic metabolic diseases suggested that a low-carbohydrate diet could contribute to oxidative stress due to nutrient imbalance, thereby increasing hypertension risk, particularly in older populations [31].

In the BLS group, both below-average and above-average intake of protein, dietary fiber, and vitamin C were associated with an increased hypertension risk. Although there was no difference in nutrient intake between groups, socioeconomically disadvantaged individuals often face a combination of factors that elevate hypertension risk, including chronic stress, limited access to healthcare services, exposure to adverse environments, and reduced social support. For example, individuals with lower socioeconomic status often experience higher levels of chronic stress due to financial instability, job insecurity, and limited social support. Chronic stress activates the hypothalamic–pituitary–adrenal axis, leading to increased cortisol production, which is strongly associated with elevated blood pressure [32,33]. A study analyzing data from the Korean Genome and Epidemiology Study found that lower income levels were significantly associated with higher hypertension incidence, particularly among women, highlighting the critical role psychosocial stressors play in hypertension development among low-income populations [34]. For instance, findings from the INTERMAP study indicated that vegetable protein intake was inversely related to blood pressure. This result is consistent with the suggestion that a diet rich in vegetables should be part of a healthy lifestyle to prevent hypertension [35]. Additionally, research by Darmon and Drewnowski (2008) demonstrated that individuals from lower socioeconomic groups often face challenges accessing diets rich in essential nutrients, such as protein, dietary fiber, and vitamins [36]. These inadequacies contribute to heightened risks of hypertension and other chronic conditions. The study underscored the complex interplay between insufficient micronutrient intake and the development of hypertension, supporting the notion that nutritional imbalances have a more pronounced impact in socioeconomically disadvantaged populations. For fat intake, both groups had a significantly higher probability of hypertension at below-average intake, but the probability ratio was much higher in the BLS group. Furthermore, low overall fat intake, common among low-income older adults, may also negatively impact blood pressure regulation. Essential fatty acids are crucial for vascular health, and their deficiency can lead to heightened susceptibility to hypertension [7,37]. These findings resonate with the observed higher odds ratio for below-average fat intake and hypertension in the BLS group, highlighting the compounded effects of poor dietary quality and metabolic stress.

The analysis of diabetes prevalence revealed a consistent association between below-average energy and fat intake and increased odds of diabetes in both groups, with a more substantial effect observed in the BLS group. This is consistent with evidence linking energy deficits and poor-quality fat intake to insulin resistance and impaired glucose metabolism, particularly in populations with limited access to balanced diets [17,38]. For protein, carbohydrates, and vitamin C, below-average intake was associated with higher odds of diabetes in both groups, suggesting that deficiencies in essential macronutrients and antioxidants contribute to poor glycemic control. Additionally, the BLS group showed higher odds of diabetes even with above-average intake of these nutrients, indicating that excessive consumption of certain foods may exacerbate metabolic risk factors in socioeconomically disadvantaged populations. This finding aligns with research showing that high-carbohydrate diets, even without overnutrition, may lead to glucose dysregulation in vulnerable groups [39]. Regarding dietary fiber, below-average intake was significantly associated with increased odds of diabetes in the BLS group but not in the non-BLS group. Dietary fiber has been shown to improve insulin sensitivity and glycemic control, and its absence in the BLS group may reflect a lack of access to high-fiber foods such as fruits, vegetables, and whole grains [18,40]. This highlights the importance of promoting fiber-rich diets, particularly in populations with socioeconomic constraints.

The results of the logistic analysis examining the relationship between hypertension and physical activity showed that sedentary behavior for more than six hours a day is associated with a high prevalence of hypertension, with the likelihood increasing significantly for those sitting for more than 12 h a day. This result is consistent with the findings of a study by Dunstan et al. (2012), which confirmed that sedentary behavior for an extended period is an independent risk factor for hypertension, regardless of the level of physical activity [41]. The increased odds in the BLS group suggest that socioeconomic constraints lead to a lack of resources or opportunities to offset sedentary time through physical activity, thereby worsening the negative effects of inactivity [26]. Adams et al. (2023) explored these associations’ physiological mechanisms, revealing that prolonged sitting impairs vascular function, leading to increased blood pressure and hypertension risk [42]. The elevated odds ratios in the BLS group may reflect biological effects and environmental factors, such as limited access to walkable spaces or health education initiatives.

A similar pattern was observed for diabetes prevalence, with sedentary time exceeding nine hours per day being associated with significantly higher odds of diabetes in both groups. The risk increased further in the BLS group, where even six to eight hours of sedentary behavior was linked to a higher prevalence. Owen et al. (2010) highlighted that prolonged sitting leads to reduced muscle activity, impairing glucose uptake and increasing insulin resistance [43]. A systematic review by Biswas et al. (2015) found that individuals with high sedentary time have a significantly elevated risk of diabetes, independent of overall physical activity [44]. This finding aligns with the observed increased odds of diabetes in the BLS group with six to eight hours of sedentary time, indicating a lower threshold for adverse effects in disadvantaged populations. Healy et al. (2008) further emphasized that breaking sedentary time with light-intensity activities, such as standing or short walks, can significantly improve glycemic control [45]. However, individuals from low-income backgrounds often face structural barriers that prevent frequent breaks from sitting, such as job-related constraints or limited access to safe spaces for physical activity.

The frequency of walking was inversely associated with the odds of hypertension and diabetes, although the strength of these associations varied between the groups. Walking less than one day per week significantly increased the odds of both conditions in both groups. However, walking two to four days per week was associated with a higher prevalence of diabetes in the BLS group. This finding may reflect the complex interplay between socioeconomic factors and physical activity patterns. According to a study by Rizka et al. (2022), walking exercise significantly reduced systolic and diastolic blood pressure and blood sugar levels in elderly participants [46]. However, the BLS group may also indicate that walking alone is insufficient to address the wide range of health issues that vulnerable populations face, such as poor nutrition, chronic stress, and difficulty accessing healthcare services [47].

The strength of this study is that it revealed significant differences in dietary intake, physical activity, and biochemical indicators between the non-BLS and the BLS groups. This study focuses on socioeconomic disparities, highlighting the combined impact of lower socioeconomic status on the increased prevalence of hypertension and diabetes, providing valuable insights into public health inequalities. In addition, the study incorporates a wide range of variables, including demographic, dietary, physical activity, and biochemical factors, to evaluate their collective impact on chronic disease prevalence. This multifaceted approach enhances the validity of the findings and offers a comprehensive understanding of the determinants of hypertension and diabetes. This study highlights the need for policy interventions to address the socioeconomic gaps that contribute to the high prevalence of chronic diseases. This is especially prevalent among individuals receiving BLS. Effective strategies should focus on improving the eating habits, reducing the sedentary lifestyles, and increasing the physical activity of vulnerable groups. For example, programs such as food subsidies or vouchers to provide fresh produce, whole grains, and low-fat protein sources for low-income populations can help alleviate the nutritional imbalances observed within the BLS group. In addition, investing in infrastructure such as safe and accessible trails, parks, and recreational facilities can promote regular physical activity. By prioritizing these policies, governments and stakeholders can reduce the economic burden of chronic diseases among socioeconomically vulnerable groups through the reduction of health disparities.

The cross-sectional design of this study limits its ability to determine causal relationships between socioeconomic status, lifestyle factors, and the prevalence of chronic diseases. The observed associations are strong, but longitudinal studies must confirm the causal relationships. The reliance on self-reported data for physical activity and dietary habits may have introduced recall bias or inaccuracies. Participants may have overestimated their activity levels or underestimated unhealthy dietary choices, potentially affecting the reliability of the results.

## 5. Conclusions

This study highlights the critical role of socioeconomic status, dietary imbalances, and low physical activity in the prevalence of hypertension and diabetes. The results of the study emphasize that people in the BLS group face a complex risk as they have limited access to nutritious food, low levels of physical activity, and a long sedentary lifestyle. It is essential to address the root causes of socioeconomic inequality, including poverty and inadequate access to healthcare, to improve health outcomes for low-income people. Policies that integrate lifestyle interventions with broader social support systems will likely have the most significant impact.

## Figures and Tables

**Table 1:** Demographic characteristics of non-BLS and BLS groups.

	Non-BLS (*n* = 13,439, 89.3%)	BLS (*n* = 1583, 10.5%)	*p*-Value
Age, years	73.6 ± 5.1	73.8 ± 4.9	0.321 ^a^
Sex, female	7453 (55.5)	1063 (67.2) ^c^	<0.001 ^b^
Systolic blood pressure, mmHg	121.5 ± 17.2	132.9 ± 17.6	<0.001 ^a^
Diastolic blood pressure, mmHg	72.5 ± 9.6	81.5 ± 10.1	<0.001 ^a^
Weight, kg	60.4 ± 10.1	58.4 ± 10.2	<0.001 ^a^
BMI, kg/m^2^	24.0 ± 3.1	24.2 ± 3.4	0.173 ^a^
Education Status			<0.001 ^b^
To elementary school	6405 (54.3)	992 (74.1) ^c^	
To middle school	1856 (15.7)	167 (12.5) ^d^	
To high school	2249 (19.1)	124 (9.3) ^d^	
Above college	1281 (10.9)	55 (4.1) ^d^	
Marital Status			<0.001 ^b^
With spouse	13,389 (99.6)	1521 (96.1) ^d^	
Not married	49 (0.4)	62 (3.9) ^c^	
Smoking History			<0.001 ^b^
Never or past	11,466 (90.7)	1268 (87.1) ^d^	
Current	1173 (9.3)	188 (12.9) ^c^	
Hypertension, yes	7088 (55.3)	942 (62.9) ^c^	<0.001 ^b^
Diabetes mellitus, yes	2782 (21.7)	414 (27.7) ^c^	<0.001 ^b^
Fasting blood glucose, mg/dL	107.7 ± 26.0	110.4 ± 32.2	<0.001 ^a^
Hemoglobin A1c, %	6.0 ± 0.8	6.1 ± 0.9	<0.001 ^a^
Total cholesterol, mg/dL	181.5 ± 39.4	180.1 ± 39.4	0.187 ^a^
Triglycerides, mg/dL	129.9 ± 77.4	134.7 ± 86.3	0.031 ^a^
Energy intake, Kcal	1637.4 ± 669.4	1397.4 ± 632.6	<0.001 ^a^
Protein, g	55.5 ± 28.5	44.9 ± 26.1	<0.001 ^a^
Fat, g	28.4 ± 21.7	21.4 ± 20.3	<0.001 ^a^
Carbohydrates, g	279.1 ± 113.3	246.7 ± 110.1	<0.001 ^a^
Dietary fiber, g	26.1 ± 14.7	20.3 ± 12.4	<0.001 ^a^
Vitamin C, mg	69.1 ± 85.3	48.5 ± 61.6	<0.001 ^a^
Sedentary Time			<0.001 ^b^
5 h or less per day	2952 (26.1)	276 (21.9) ^d^	
6–8 h per day	3206 (28.4)	326 (25.9) ^d^	
9–11 h per day	2746 (24.3)	267 (21.2) ^d^	
12 h or more per day	2403 (21.3)	392 (31.1) ^c^	
Walking habits			<0.001 ^b^
None or 1 day per week	3458 (29.4)	510 (38.2) ^c^	
2–4 days per week	2998 (25.5)	323 (24.2)	
5 days or more per week	5299 (45.1)	501 (37.6) ^d^	

Values are presented as mean ± SD or *n* (%). BLS, Basic Livelihood Security; BMI, body mass index. ^a^ *p*-value obtained by Student’s *t*-test. ^b^ *p*-value obtained by Pearson’s chi-square test. ^c^ Statistically significant association by adjusted standardized residual > 1.96 (*p* < 0.05). ^d^ Statistically significant association by adjusted standardized residual < −1.96 (*p* < 0.05).

**Table 2:** The odds ratio of hypertension for nutrients intake.

Variables	Classification	Non-BLS OR of Hypertension (CI)	BLS OR of Hypertension (CI)
Energy intake	Above average	1	1.197 (0.973–1.471)
	Below average	1.056 (0.972–1.146)	1.346 (1.148–1.578)
Protein	Above average	1	1.331 (1.051–1.685)
	Below average	1.063(0.978–1.156)	1.294 (1.110–1.509)
Fat	Above average	1	1.279 (0.989–1.654)
	Below average	1.102 (1.012–1.200)	1.362 (1.169–1.586)
Carbohydrates	Above average	1	1.081 (0.896–1.304)
	Below average	1.037 (0.957–1.124)	1.441 (1.221–1.702)
Dietary fiber	Above average	1	1.270 (1.051–1.535)
	Below average	1.060 (0.976–1.151)	1.300 (1.104–1.532)
Vitamin C	Above average	1	1.346 (1.044–1.736)
	Below average	1.040 (0.972–1.163)	1.325 (1.139–1.540)

BLS, Basic Livelihood Security; OR, odds ratio; CI, confidence interval. Adjusted variables: sex, education status, smoking history.

**Table 3:** The odds ratio of diabetes mellitus for nutrient intake.

Variables	Classification	Non-BLS OR of Diabetes (CI)	BLS OR of Diabetes (CI)
Energy intake	Above average	1	1.260 (0.997–1.592)
	Below average	1.208 (1.095–1.334)	1.553 (1.307–1.845)
Protein	Above average	1	1.423 (1.100–1.842)
	Below average	1.126 (1.018–1.245)	1.401 (1.182–1.661)
Fat	Above average	1	1.283 (0.960–1.715)
	Below average	1.115 (1.005–1.236)	1.443 (1.219–1.708)
Carbohydrates	Above average	1	1.243 (1.004–1.538)
	Below average	1.150 (1.045–1.265)	1.511 (1.265–1.804)
Dietary fiber	Above average	1	1.344 (1.094–1.650)
	Below average	0.936 (0.849–1.033)	1.209 (1.010–1.446)
Vitamin C	Above average	1	1.479 (1.119–1.954)
	Below average	1.184 (1.070–1.309)	1.461 (1.235–1.728)

BLS, Basic Livelihood Security; OR, odds ratio; CI, confidence interval. Adjusted variables: sex, education status, smoking history.

**Table 4:** The odds ratio of hypertension for physical activity.

Variables	Classification	Non-BLS OR of Hypertension (CI)	BLS OR of Hypertension (CI)
Sedentary time per day	5 h or less	1	1.235 (0.958–1.593)
	6–8 h	1.132 (1.022–1.253)	1.434 (1.129–1.822)
	9–11 h	1.259 (1.131–1.400)	1.420 (1.092–1.847)
	12 h or more	1.240 (1.109–1.387)	1.542 (1.230–1.933)
Number of walking days per week	5 days or more	1	1.140 (0.903–1.438)
	2–4 days	1.038 (0.947–1.137)	1.321 (1.090–1.601)
	1 day or less	1.131 (1.033–1.238)	1.510 (1.237–1.842)

BLS, Basic Livelihood Security; OR, odds ratio; CI, confidence interval. Adjusted variables: sex, education status, smoking history.

**Table 5:** The odds ratio of diabetes mellitus for physical activity.

Variables	Classification	Non-BLS OR of Diabetes (CI)	BLS OR of Diabetes (CI)
Sedentary time per day	5 h or less	1	1.160 (0.973–1.427)
	6–8 h	1.133 (0.998–1.287)	1.407 (1.072–1.846)
	9–11 h	1.308 (1.149–1.490)	1.336 (1.070–1.803)
	12 h or more	1.438 (1.257–1.644)	2.045 (1.614–2.591)
Number of walking days per week	5 days or more	1	1.101 (0.958–1.532)
	2–4 days	1.100 (0.985–1.228)	1.374 (1.060–1.781)
	1 day or less	1.138 (1.023–1.267)	1.469 (1.190–1.813)

BLS, Basic Livelihood Security; OR, odds ratio; CI, confidence interval. Adjusted variables: sex, education status, smoking history.

## Data Availability

This study analyzed data released from government agencies: [https://knhanes.kdca.go.kr] (accessed on 15 October 2024).
